# Examining COVID-19 vaccination coverage and acceptability among migrants in transit through Mexico during 2021–2022: Insights from shelter surveys and interviews

**DOI:** 10.1371/journal.pone.0324325

**Published:** 2025-07-11

**Authors:** René Leyva-Flores, Belkis Aracena-Genao, Christine Allen, Diana Gómez-López, Ietza Bojórquez-Chapela, Ricardo Cortés-Alcalá

**Affiliations:** 1 Center for Research in Health Systems (CISS), National Institute of Public Health (INSP), Cuernavaca, Morelos, Mexico; 2 Center for Research in Nutrition and Health (CINyS), National Institute of Public Health (INSP), Cuernavaca, Mexico; 3 Independent Researcher, Austin, Texas, United States of America; 4 Independent Researcher, Mexico City, Mexico; 5 El Colegio de la Frontera Norte, Ensenada, Baja California, Mexico; 6 Ministry of Health (SSA), Mexico City, Mexico; Facultad Latinoamericana de Ciencias Sociales Mexico, MEXICO

## Abstract

Mexico faced significant obstacles in achieving COVID-19 vaccination coverage for migrants, particularly irregular migrants, asylum seekers, and refugees. In other contexts, migrant vaccination coverage has been influenced by prioritization policies, identification requirements, and various sociodemographic, migratory, and health factors, though these had not been fully explored in Mexico. This study analyzed factors associated with COVID-19 vaccine coverage among migrants in transit through Mexico. From November 2021 to May 2022, a convenience sample of 2,355 migrants across six shelters was surveyed on vaccination history and acceptance, as well as sociodemographic, migration, and health characteristics. To complement the survey, semi-structured interviews with migrants and key informants explored access barriers and attitudes toward COVID-19 vaccination. Of respondents, 61.1% reported receiving at least one COVID-19 vaccine dose, with 67.6% vaccinated prior to arriving in Mexico. Factors associated with vaccination included education level, country of prior residence, history of COVID-19 infection (OR = [95% CI, 1.081–1.737]), and COVID-19 testing history (OR = [95% CI, 3.825–5.999]). Among unvaccinated respondents, 81.4% expressed willingness to vaccinate, often viewing it as “protection against complications or death” and a “requirement for movement between countries.” Among respondents, 7.2% expressed concerns related to misinformation. Findings suggest that while many migrants were vaccinated before arrival, inconsistent ID requirements and age-based restrictions impeded coverage within Mexico. Addressing misinformation and aligning local practices with national policies could improve vaccine access for this population.

## Introduction

Early in the COVID-19 pandemic, the international community established a goal to vaccinate 70% of the world’s population by mid-2022, recognizing that widespread immunization was essential to controlling the virus’s spread [1]. Europe [2] and the United States [3] reached this target nearly a year before the deadline [1], while by 2025, only 14 out of 54 African countries had met the goal [4]. As of January 2024, the average vaccination rate in Latin America stood at 70.4%, with significant variation across the region, from 92.2% in Chile to just 3.2% in Haiti [5]. Mexico achieved a coverage rate of 62.4% almost three years after launching its COVID-19 vaccination campaign [5,6], although disparities were observed across age groups [7] and states [6].

These differences among countries in reaching vaccination targets were associated, among other factors, with vaccine availability and acceptance levels. Differences in vaccine availability across countries were stark. High-income countries, such as the United States, United Kingdom, European Union, and Japan, secured a combined 1.3 billion vaccine doses by November 2020, allowing them to vaccinate large portions of their populations early in the pandemic. In contrast, low- and middle-income countries faced significant limitations in accessing doses. Efforts were made to provide 2 billion doses to 92 low- and middle-income countries by the end of 2021, yet vaccine availability in these regions lagged considerably, underscoring a persistent global disparity [8]. Differences in acceptance rates were also observed, with 2021 estimates ranging from 51.6% in Russia to 97.6% in China [9]. For this study’s purposes, it is noteworthy that the acceptance rate in the United States was 66.6%, compared to 81.2% in Mexico [9].

In addition to cross-country differences, within-country disparities also affect vulnerable groups, such as migrant populations [10]. These groups often face barriers [11] to accessing health services that are comprehensive, appropriate, timely, and of high quality [12]. According to the United Nations High Commissioner for Refugees (UNHCR), while 162 countries included refugees, asylum seekers, and displaced persons in their vaccination programs, only 72 countries (44%) had actually started administering COVID-19 vaccinations to these groups, and no data were available on the extent of coverage achieved [13]. Other reports indicate that just 10% (19 of 185 countries) prioritized migrant populations in their vaccination plans [14]. For these groups, vaccination coverage reported in 2022 was 45.8% in Iraq [15], 66% in Pakistan [16], and 75% in the United Kingdom [17].

Regarding vaccine acceptance rates among the general population across countries, proportions ranged from 21.9% in the United Kingdom to 88.5% in Japan [18]. These differences may have been linked to a range of factors whose influence varied by country, including an “infodemic” of misinformation [19], the rapid timeline of vaccine development [20] and the resulting uncertainties about vaccine efficacy and safety [21]. Evolving public health guidelines likely played a role as well; for instance, early guidance recommended using the same type and brand of vaccine for COVID-19 immunization [22,23], while later evidence highlighted the potential benefits of vaccine interchangeability [24,25]. Additionally, religious practices [18] and beliefs downplaying the severity of COVID-19 [26] also contributed to differences in vaccine acceptance.

In Mexico, additional factors compounded the challenges to achieving vaccine coverage for migrants, especially irregular migrants, asylum seekers, and refugees. Key among these were federal prioritization policies, which, consistent with other countries, prioritized older adults and individuals with preexisting conditions that elevate the risk of severe COVID-19 outcomes (e.g., cancer, HIV, diabetes, hypertension) [27]. On the ground, however, vaccine providers often required identification documents or proof of residence [28,29] in contrast to federal regulations stating that official ID should not be required to receive the vaccine [30]. These combined factors largely excluded migrant populations, who tend to be younger and generally in good health, though many may have weakened immune responses due to the hardships of migration [31]. Additionally, most migrants did not possess official identification documents [32,33].

Given these regulatory and administrative barriers, along with individual healthcare-seeking behaviors, COVID-19 vaccine acceptability and access among migrants were likely to be impacted. However, the extent of their impact was unknown, motivating the present study, conducted during the third (June to October 2021) and fourth (December 2021 to February 2022) COVID-19 waves in Mexico. The study’s objective was to analyze, using qualitative and quantitative methods, the factors associated with COVID-19 vaccine coverage among migrants in transit through Mexico.

## Materials and methods

### Study design and Setting

**Fig 1:**
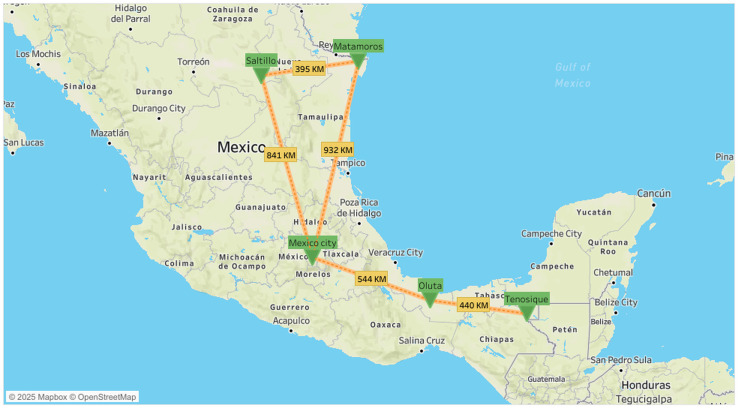
Geographic Distribution of Surveyed Migrant Shelters Along the Gulf Migration Route in Mexico.

This study utilized a cross-sectional design framed by a complementarity approach [34,35], wherein qualitative data were used to contextualize and interpret quantitative findings. Fieldwork was carried out from November 2021 to May 2022, during the third and fourth COVID-19 waves, at six migrant shelters (*Casas del Migrante*) located in five Mexican cities with high international mobility. Three data collection components were implemented: (1) a quantitative survey with migrants to assess vaccination uptake, (2) semi-structured interviews with migrants to explore individual experiences and attitudes toward vaccination, and (3) interviews with key informants involved in migrant health and support services to understand systemic barriers and facilitators to vaccine access. The quantitative research question asked, “What are the sociodemographic, health, and migration-related factors associated with COVID-19 vaccination uptake among migrants in transit through Mexico?” The qualitative research question examined, “What are the perceived barriers and facilitators to COVID-19 vaccine access and acceptance among migrants in transit through Mexico, as described by migrants and key informants?” Study sites included Tenosique (Tabasco), a critical crossing near the Guatemala border, Oluta (Veracruz), Mexico City, Saltillo (Coahuila), and Matamoros (Tamaulipas), one of the major crossing points into the United States (Fig 1).d

### Sample size and participants

A convenience sample of migrant shelters were selected based on their location along the “Gulf Migration Route,” which is the shortest land route between Mexico and the United States. Other selection criteria included the presence of governmental agencies, local NGOs, and international cooperation institutions, as well as the shelter’s extensive experience in serving migrant populations (participating shelters averaged 10 years of service provision).

For the quantitative survey, eligible participants were migrants aged 18 and older (or 12–17 for unaccompanied minors) residing at one of the selected shelter sites at the time of data collection. A target sample size of 3,000 migrants was set, with an average of 600 per shelter, except for the two shelters in Mexico City, where 300 surveys were targeted at each location. This sample size was determined based on available resources and each shelter’s reported average monthly migrant intake over the previous year. However, due to fluctuating migration patterns during the study period, extended shelter stays, and COVID-19 entry restrictions, the target sample size was not reached. A total of 2,552 migrants were invited to participate in the survey. Of these, 2,355 (92.3%) agreed to participate, 18 (0.7%) declined, and 179 (7.0%) started but chose not to complete the survey.

The qualitative component included a purposive sampling of 32 migrants and 27 key informants. Migrants were selected to capture diverse demographic perspectives (e.g., women, pregnant women, men, LGBTQIA+ individuals). Eligibility criteria for migrants in this component were the same as for the quantitative survey, with the additional requirement of not having participated in the survey. Budget limitations restricted qualitative interviews to three of the six selected shelters, specifically those at strategic points along the migration route: Tenosique (southern border), Acayucan (central transit), and Matamoros (northern border).

Migrant participants were recruited in collaboration with shelter directors, who invited eligible individuals to participate.

Key informants were selected based on their expertise in migrant health and mobility services, providing professional insights into factors influencing COVID-19 vaccination among migrants. In collaboration with local health authorities, a list of 72 potential participants was compiled using inclusion criteria such as prior experience with migrant populations, roles in healthcare policy implementation or evaluation, involvement in government or civil society organizations supporting in-transit migrants, or technical roles in local health services. Of these, 27 agreed to participate, and all completed the interview. Each key informant was individually contacted with a personalized document explaining the study’s purpose, describing the interview content, and emphasizing the voluntary nature of participation.

### Data collection

#### Quantitative survey.

To collect data on the health risks, conditions, and COVID-19 vaccination coverage of migrants in transit through Mexico, a 25-minute electronic questionnaire (see supplementary material 1. Questionnaire) was administered by trained migrant shelter staff at each site using the Research Electronic Data Capture (REDCap) platform. The survey, conducted in Spanish, included nine modules covering sociodemographic characteristics, health conditions, COVID-19 status, access to health services, mobility patterns, COVID-19 vaccination prevalence, and vaccine acceptability factors.

The survey was administered by shelter staff directly involved in psychosocial or health support services (e.g., social workers, sociologists, anthropologists, nursing staff) who had extensive experience working with vulnerable migrant populations. Staff received training on the data collection platform, survey content, and ethical standards, although their daily work in the shelters also provided them with a strong awareness of the vulnerabilities faced by migrants.

To ensure data quality and adherence to ethical principles—particularly voluntary participation, confidentiality, privacy, and anonymity—data collection was continuously monitored. Monitoring involved weekly virtual meetings, phone check-ins as needed, and three on-site visits over the course of the project. This process included identifying and addressing challenges related to survey goals, as well as analyzing a randomly selected sample of surveys (12 weekly, two per shelter) to detect and resolve inconsistencies.

### Qualitative interviews

The primary objective of the semi-structured interviews was to capture the insights and experiences of both migrants and key informants by exploring areas of alignment with the quantitative data. This approach was intended to offer a more comprehensive understanding of the interplay between mobility and health risks within the context of the COVID-19 pandemic. Similar semi-structured interview guides were used for each group (in-transit migrants and key decision-makers), structured around five central themes: dynamics of mobility and migration, COVID-19, health risks and problems, access to health services, and protective factors along the migratory route. However, these topics were adapted to reflect the unique perspectives and experiences of each group. The interview guide, codebook, and qualitative outputs from the study are available in the supplementary materials (2. Interview guide, 3. Codebook, 4. Qualitative outputs).

Interviews were audio-recorded and conducted face-to-face by a team of three professionals specializing in social sciences and health, with extensive experience in migration and international mobility. These team members were involved throughout the research process, from study design to data collection and analysis.

### Analysis

#### Variables.

The primary variables of interest were vaccination coverage and vaccine acceptance. Vaccination coverage was defined as the uptake of at least one dose of the COVID-19 vaccine, while vaccine acceptance was defined as willingness to be vaccinated, analyzed among participants who had not yet received any COVID-19 vaccine doses. Additional variables included sociodemographic characteristics (age, sex, gender identity, education, marital status, ethnic group, and parenthood status), survey location, international mobility and migration factors (last country of residence, migratory status, and traveling with a minor), health status (preexisting morbidities, history of COVID-19, and self-reported health status), and COVID-19 risk perception (measured by the survey question, “Have you had a COVID-19 test?”). The variable “sex” referred to biological sex assigned at birth, while “gender” was understood as a social construct, aimed at exploring participants’ gender identity.

### Quantitative analysis

A descriptive analysis was performed on sociodemographic characteristics, mobility and migration patterns, health status, and COVID-19 risk perception, comparing vaccinated and unvaccinated participants. This phase also examined differences between those vaccinated in Mexico versus abroad and explored reasons for vaccine refusal among participants unwilling to be vaccinated despite availability. Proportional differences were analyzed using the Chi-square test, while differences in age and years of education were assessed using the Mann-Whitney U test due to non-normal distribution [36].

A bivariate analysis (see supplementary material 5. Bivariate analysis) was conducted to identify factors significantly associated with vaccination status, which informed the selection of variables for the regression model. The multivariable logistic regression model used vaccination status as the response variable (1 = yes, 0 = no), with predictor variables including factors statistically significant in the bivariate analysis (p < 0.05) and theoretically linked to vaccination likelihood. Education was categorized as no schooling, 1–6 years, 7–14 years, and ≥15 years. The last country of residence before arriving in Mexico was categorized as Honduras, Guatemala, Nicaragua, El Salvador, Chile, or Other. Additional variables were COVID-19 history, testing status, and survey location (Matamoros, Mexico City, Oluta, Saltillo, and Tenosique).

Migration status was excluded from the final model due to high correlation with other explanatory variables, such as education and country of residence. Sex and gender were also excluded from the multivariable model, as they showed no statistically significant relationship with vaccination status in the bivariate analysis. Model fit was assessed using the Akaike Information Criterion (AIC) [37,38] with additional goodness-of-fit tests conducted via Pearson’s Chi-square and the Hosmer-Lemeshow test [39]. All statistical analyses were performed using the statistical software for data science, Stata version 15.

### Qualitative analysis

Audio recordings from the interviews were processed and coded by three analysts using AtlasTi-V9.1 software under the supervision of the principal investigator. A deductive analytic approach was applied, focusing on the five central themes explored in the interviews. Codes were organized into a codebook, which served as a reference for systematically categorizing content and constructing matrices to synthesize interviewee testimonies. This coding process was carried out collaboratively by the same analysts.

The analysis concentrated on two primary areas: (1) conditions influencing vaccine access, including barriers and facilitators (e.g., national regulations, institutional implementation strategies, social support networks, vaccine availability, and vaccine types), and (2) reasons for COVID-19 vaccine acceptance or rejection. Interviews with in-transit migrants also explored experiences, perceptions, and opinions related to these themes in their countries of origin and transit before arriving in Mexico. These interviews provided insights into lived experiences and the vaccination process in varied settings, capturing themes that are challenging to address with quantitative methods.

To minimize researcher bias in the qualitative analysis, several procedures were implemented. First, researchers collaboratively developed the codebook based on the thematic categories in the interview guide. Second, the three interviewers held regular meetings to reach consensus on responses that were challenging to categorize. Third, six interviews were randomly selected and independently coded by the interviewers. The principal investigator reviewed these independently coded interviews to assess consistency with the predefined codes. Finally, new codes corresponding to emergent themes not covered in the interview guide were identified and jointly defined by the interviewers and the principal investigator.

### Integration of quantitative and qualitative information

Quantitative and qualitative data were collected simultaneously, followed by an analysis aimed at identifying areas of coherence between the two data types [40]. The integration of the quantitative and qualitative data involved a bidirectional, complementary analysis, wherein independently processed data were analytically integrated. The integration fit was assessed by comparing qualitative testimonials with quantitative results [41], allowing qualitative insights to complement statistical findings related to vaccine coverage and acceptability.

This analytical integration highlighted both convergences and divergences between the magnitudes of identified factors and experiential perspectives. Through this process, the combined data offered a more nuanced understanding of the factors influencing vaccine access and acceptance. To mitigate potential biases [42], each phase of the study—from data collection through interpretation—was carefully designed and implemented, with attention to the ethical handling, storage, processing, and analysis of data.

### Ethics approval and consent to participate

Shelter staff involved in the study informed participants, both collectively and individually, about the project’s objectives. All shelter residents were advised that the study aimed to generate information to support decision-making and improve responses to their health needs. They were also informed that, under Mexico’s General Health Law, all individuals on Mexican territory, regardless of migratory status, have the right to access government health services [43].

Shelter residents were then invited to participate, and it was stressed that participation was entirely voluntary, and that no personally identifiable information would be collected. Participants were further informed that surveys and interviews would be conducted privately, with shelter staff directly involved to ensure confidentiality. It was clarified that participants were free to skip any questions or end the survey or interview at any point if they wished.

All participants were verbally informed that their decision to participate, or not participate, in the study would not affect their access to services or treatment provided by the migrant shelter or other institutions supporting migrants in transit through Mexico. To ensure privacy and anonymity, verbal consent was obtained prior to administering surveys and interviews. Verbal consent is recommended when gathering information from groups experiencing high social vulnerability, such as irregular migrants, who often live under conditions of uncertainty, fear, and threats [44,45]. This verbal consent process was documented electronically, with consent instructions displayed on the first screen of the data entry form (see supplementary material 6. Verbal consent -shoot screen of the data entry form). The interviewer read these instructions aloud, and if the participant consented, they clicked the designated consent field, eliminating the need for physical signatures. This consent procedure was reviewed and approved by the Ethics Subcommittee of El Colegio de la Frontera Norte, with approval renewed annually (2021–2022, Approval Number 079_230821) (see supplementary material 7. IRB approval Verbal consent).

All key informants interviewed signed written informed consent. Interviews were conducted in private spaces to maintain confidentiality, and an alphanumeric code was assigned to all audio recordings and notes to ensure anonymity. No personal information about decision-makers or their organizations was recorded.

## Results

### Sociodemographic, migration, and health characteristics

The study sample’s sociodemographic, migration, and health characteristics are outlined in Table 1, stratified by vaccination status. Of the total survey respondents, 61.1% were vaccinated. The mean age of participants was 28.1 (SD = 9.7). Most respondents (87.4%) reported attending 1–14 years of school, and 7.3% had completed higher education (≥ 15 of schooling). The majority (71.6%) were assigned male at birth, though a smaller proportion (66.3%) currently identified as male. Overall, 59.1% of respondents were single, and 47.5% reported having children. A minority identified as belonging to ethnic groups, with 7.4% identifying as Indigenous and 9.6% as Afro-descendant.

**Table 1:** Sociodemographic, Migration, and Health Characteristics of Migrants in Mexico by COVID-19 Vaccination Status, November 2021–May 2022.

Category	Variable	Total (n = 2,355)	Vaccinated (n = 1,439; 61.1%)	Unvaccinated (n = 916; 38.9%)	*p* value
Sociodemographic Characteristics	Age (years): Mean (SD)	28.1 (9.7)	28.9 (9.6)	26.8 (9.7)	0.00*
	Education (years): Mean (SD)	8.0 (4.0)	8.2 (4.1)	7.7 (3.9)	0.00*
		**n (% of Total)**	**n (% of vaccinated)**	**n (% of unvaccinated)**	
	Education Level				0.00*
	No school	125 (5.3)	60 (48.0)	65 (52.0)	
	1–6 years	925 (39.3)	563 (60.9)	362 (39.1)	
	7–14 years	1,133 (48.1)	696 (61.4)	437 (38.6)	
	15 and over	172 (7.3)	120 (69.8)	52 (30.2)	
	Sex at Birth				0.94
	Male	1,687 (71.6)	1,030 (61.1)	657 (38.9)	
	Female	668 (28.4)	409 (61.2)	259 (38.8)	
	Self-Reported Gender				0.69
	Male	1,562 (66.3)	967 (61.9)	595 (38.1)	
	Female	701 (29.8)	417 (59.5)	284 (40.5)	
	LGBTQIA+	83 (3.5)	49 (59.0)	34 (41.0)	
	No Response	9 (0.4)	6 (66.7)	3 (33.3)	
	Marital Status				0.09+
	Has Partner	963 (40.9)	608 (63.1)	355 (36.9)	
	No Partner	1,392 (59.1)	831 (59.7)	561 (40.3)	
	Has Children				0.00*
	Yes	1,118 (47.5)	743 (66.5)	375 (33.5)	
	No	1,237 (52.5)	696 (56.3)	541 (43.7)	
	Ethnic Group				0.00*
	Indigenous	174 (7.4)	90 (51.7)	84 (48.3)	
	Afro-descendent	225 (9.6)	166 (73.8)	59 (26.2)	
	No	1,956 (83.1)	1,183 (60.5)	773 (39.5)	
International Mobility and Migration	Last Country of Residence				0.00*
	Honduras	1,426 (60.6)	817 (57.3)	609 (42.7)	
	Guatemala	223 (9.5)	116 (52.0)	107 (48.0)	
	Nicaragua	172 (7.3)	129 (75.0)	43 (25.0)	
	El Salvador	146 (6.2)	84 (57.5)	62 (42.5)	
	Chile	112 (4.8)	92 (82.1)	20 (17.9)	
	Other Countries^1^	276 (11.7)	201 (72.8)	75 (27.2)	
	Migratory Status				0.00*
	Irregular	1,594 (67.7)	933 (58.5)	661 (41.5)	
	Asylum Seeker	584 (24.8)	408 (69.9)	176 (30.1)	
	Refugee	156 (6.6)	84 (53.9)	72 (46.2)	
	Visa/Permit	21 (0.9)	14 (66.7)	7 (33.3)	
	Female w/ Minor (<18)				0.61
	Yes	309 (46.3)	186 (60.2)	123 (39.8)	
	No	359 (53.7)	223 (62.1)	136 (37.9)	
	Male w/ Minor (<18)				0.63
	Yes	185 (11.0)	116 (62.7)	69 (37.3)	
	No	1,502 (89.0)	914 (60.9)	588 (39.2)	
Health Status	Self-Reported Health				0.02^†^
	Good or Excellent	1,848 (78.5)	1,148 (62.1)	700 (37.9)	
	Average	470 (20.0)	264 (56.2)	206 (43.8)	
	Bad or Very Bad	37 (1.6)	27 (73.0)	10 (27.0)	
	Pregnancy Status (among females at birth: n = 668)				0.01^†^
	Yes	41 (6.1)	23 (56.1)	18 (43.9)	
	No	597 (89.4)	375 (62.8)	222 (37.2)	
	Unknown	30 (4.5)	11 (36.7)	19 (63.3)	
	Preexisting Chronic Condition				0.09+
	Yes	340 (14.4)	222 (65.3)	118 (34.7)	
	No	2,015 (85.6)	1,217 (60.4)	798 (39.6)	
	History of COVID				0.00*
	Yes	542 (23.0)	366 (67.5)	176 (32.5)	
	No	1,792 (76.1)	1,065 (59.4)	727 (40.6)	
	No Response	21 (0.9)	8 (38.1)	13 (61.9)	
Risk Perception	Has taken a COVID test				0.00*
	Yes	1,437 (61.0)	1,067 (74.3)	370 (25.8)	
	No	918 (39.0)	372 (40.5)	546 (59.5)	
Transit Community	Survey Location				0.00*
	Tenosique, Tabasco^Y^	597 (25.4)	253 (42.4)	344 (57.6)	
	Oluta, Veracruz^X^	388 (16.5)	330 (85.1)	58 (15.0)	
	Mexico City^V^	361 (15.3)	281 (77.8)	80 (22.2)	
	Saltillo, Coahuilat	542 (23.0)	326 (60.2)	216 (39.9)	
	Matamoros, Tamaulipas^∞^	467 (19.8)	249 (53.3)	218 (46.7)	

^1^Includes Venezuela, Cuba, and other countries

Statistical significance: * p = 0.00 [CI: 99%]; ^†^ p ≤ 0.05 [CI: 95%]; + p ≤ 0.10 [CI: 90%]

^Y^Entry point: southern border; ^X^Connection point; ^v^Central hub; ^t^Connection point; ^∞^Exit point: northern border

Most participants (83.5%) reported their last country of residence as being within Central America, with the majority from Honduras (60.6%). Chile was among the five most frequently reported last countries of residence, listed by 4.8% of respondents, all of whom were of Haitian origin. With respect to migratory status, the largest group (67.7%) consisted of irregular migrants, while 24.8% were asylum seekers and 6.6% were refugees. The remaining 0.9% held official documentation such as a visa or permit. Among the female participants (n = 668), 46.3% reported traveling with minors, compared to 11.0% of male participants (n = 1,687).

In terms of health status, 78.5% of respondents considered their health to be good or excellent. Of all female respondents, 6.1% reported being pregnant. Additionally, 14.4% of all respondents indicated having at least one preexisting chronic health condition. With respect to COVID-19 history, 23.0% reported having previously contracted COVID-19, and 61.0% had taken a COVID-19 test. Of the total surveyed, 25.4% of participants were interviewed in Tenosique, Tabasco.

### Comparison of Vaccinated and Unvaccinated Groups

The comparison between vaccinated and unvaccinated groups revealed statistically significant differences across most analyzed variables. Vaccinated participants had a mean age of 28.9 years, compared to 26.8 years for unvaccinated participants. Regarding education, 69.8% of those who reported having 15 or more years of schooling were vaccinated; in comparison only 48% of those who had not attended school were vaccinated. A higher proportion of vaccinated individuals were in a relationship (63.1%) and had children (66.5%). Additionally, vaccinated individuals were more likely to identify as Afro-descendant (73.8%), report Chile as their last country of residence (82.1%), and have asylum-seeker status (69.9%). Vaccinated respondents were also more likely to rate their health as bad or very bad (73.0%), report a preexisting chronic health condition (65.3%), have had COVID-19 (67.5%), and have taken a COVID-19 test (74.3%). Among vaccinated women, 62.8% reported not being pregnant. Participants surveyed in Oluta, Veracruz, reported the highest frequency of vaccination (85.1%) compared to other survey locations. No significant differences were found between vaccinated and unvaccinated groups regarding sex, gender identity, or whether participants were traveling with minors.

Data from surveys and interviews highlighted barriers like regulatory age restrictions and administrative ID requirements, as well as several facilitators such as local shelter management and perceptions about the benefits of the vaccine that influenced COVID-19 vaccine access among migrants.

Migrants and key informants identified age-based prioritization as a regulatory barrier to vaccination. Under Mexico’s COVID-19 vaccination rollout plan, only certain high-risk age groups were prioritized, which left many younger migrants ineligible for vaccination despite their interest in being vaccinated. One migrant commented, “With [the children], we did have trouble. We haven’t been able to get them vaccinated. When we’ve gone, they’ve told us that [the vaccines] are only for children that have diseases or are sick.” (15-M-CMM-TA). A local key informant reinforced this, saying, “We’ve had [vaccine] applications at the health center, and the only requirement is the age criteria.” (18-TD-SSM-TAM). As a result, key informants noted that migrants transiting through Mexico during rollout phases not aligned with their age group were ineligible for COVID-19 vaccination.

Additionally, there were conflicting reports on the possibility of having an administrative barrier to receiving the vaccine. Some migrants reported receiving support in accessing vaccination even without formal identification. For example, one migrant shared, “Well, since I didn’t have my papers, they made me one, with the second dose. Yeah […] the certificate from COMAR [Mexican Commission for Refugee Assistance], my CURP [Unique Population Registry Code], and they accepted a copy.” (07-M-CMT-TA).

However, local key informants provided mixed reports on whether migrants without identification documents could access vaccination. This divergent data is demonstrated through the following examples. One key informant explained, “[…] When the population is still being processed and they still don’t have a CURP, we have to make a temporary register so the vaccination team can record them in their system […] my role is to quickly channel the process so no situation arises that could keep someone from being vaccinated.” (16-TD-SBSO-V), but another reported, “[…] what they told us is that it [vaccination campaigns] was also for refugee applicants (not irregular migrants) that are well identified.” (16-TD-OCI-V).

Local shelter management was a notable facilitator, with directors coordinating group vaccination sessions. One shelter director explained, “Before they came here to vaccinate, […] they notified us, we coordinated, and we took the entire group to get vaccinated. […] the Wellbeing Coordinator helped us organize everyone so they could receive their vaccines.” (01-TD-CM-OL).

Regarding their perceptions of the COVID-19 vaccine’s protection and safety, one migrant shared, “Well, it’s to protect ourselves. I think it’s excellent. It’s great because, well, it’s for our health.” (11-M-CMM-TAM). Key informants echoed this, with one stating, “COVID-19 is affecting entire families, so vaccination is being fully accepted by migrants […]” (19-TD-SST-TA). On the vaccine’s effectiveness, another key informant commented, “I think they [vaccines] help a great deal because there are a lot of people that recovered with the shot. There are others that have had it [COVID-19] bad, but they always get better” (02-M-CMOA-V).

Table 2 presents the characteristics of vaccinated migrants based on whether they were vaccinated in Mexico or another country. Among vaccinated participants (n = 1,439), the majority (67.6%) received their vaccination outside of Mexico prior to arriving. Statistically significant differences were observed between those vaccinated in Mexico and those vaccinated abroad across most analyzed variables. A greater proportion of those vaccinated outside Mexico were male (71.2%) and identified as LGBTQIA+ (75.5%). Among those vaccinated abroad, a higher proportion reported their last country of residence as Nicaragua (82.2%) and held irregular migratory status (75.9%). In terms of health status, a greater percentage of those vaccinated abroad self-reported regular health (68.2%), had previously contracted COVID-19 (72.1%), and had not taken a COVID-19 test (72.9%). By survey location, Saltillo, Coahuila, had the highest proportion of participants vaccinated outside of Mexico (77.6%).

**Table 2:** Sociodemographic, Migration, and Health Characteristics of Migrants Vaccinated in Mexico vs. Abroad, October 2021–May 2022.

Category	Variable	Total (n = 1,439)	Vaccinated in Mexico (n = 466)	Vaccinated abroad (n = 973)	*p* value
Sociodemographic Characteristics	Age (years): Mean (SD)	28.9 (9.6)	29.5 (10.2)	28.6 (9.2)	0.27
	Education (years): Mean (SD)	8.2 (4.1)	8.1 (3.9)	8.2 (4.1)	0.41
		**n (% of Total)**	**n (% Vaccinated in Mexico)**	**n (% Vaccinated abroad)**	
	Education Level				0.41
	No school	60 (4.2)	15 (25.0)	45 (75.0)	
	1–6 years	563 (39.1)	194 (34.5)	369 (65.5)	
	7–14 years	696 (48.4)	219 (31.5)	477 (68.5)	
	15 and over	120 (8.3)	38 (31.7)	82 (68.3)	
	Sex at Birth				0.00*
	Male	1,030 (71.6)	297 (28.8)	733 (71.2)	
	Female	409 (28.4)	169 (41.3)	240 (58.7)	
	Self-Reported Gender				0.00*
	Male	967 (67.2)	287 (29.7)	680 (70.3)	
	Female	417 (29.0)	166 (39.8)	251 (60.2)	
	LGBTQIA+	49 (3.4)	12 (24.5)	37 (75.5)	
	No Response	6 (0.4)	1 (16.7)	5 (83.3)	
	Marital Status				0.92
	Has Partner	608 (42.3)	196 (32.2)	412 (67.8)	
	No Partner	831 (57.7)	270 (32.5)	561 (67.5)	
	Has Children				0.97
	Yes	743 (51.6)	241 (32.4)	502 (67.6)	
	No	696 (48.4)	225 (32.3)	471 (67.7)	
	Ethnic Group				0.4
	Indigenous	90 (6.3)	24 (26.7)	66 (73.3)	
	Afro-descendent	166 (11.5)	58 (34.9)	108 (65.1)	
	No	1,183 (82.2)	384 (32.5)	799 (67.5)	
International Mobility and Migration	Last Country of Residence				0.00*
	Honduras	817 (56.8)	283 (34.6)	534 (65.4)	
	Guatemala	116 (8.1)	46 (39.7)	70 (60.3)	
	Nicaragua	129 (9.0)	23 (17.8)	106 (82.2)	
	El Salvador	84 (5.8)	41 (48.8)	43 (51.2)	
	Chile	92 (6.4)	20 (21.7)	72 (78.3)	
	Other Countries^1^	201 (14.0)	53 (26.4)	148 (73.6)	
	Migratory Status				0.00*
	Irregular	933 (64.8)	225 (24.1)	708 (75.9)	
	Asylum Seeker	408 (28.4)	179 (43.9)	229 (56.1)	
	Refugee	84 (5.8)	60 (71.4)	24 (28.6)	
	Visa/Permit	14 (1.0)	2 (14.3)	12 (85.7)	
	Female w/ Minor (<18)				0.67
	Yes	186 (12.9)	79 (42.5)	107 (57.5)	
	No	223 (15.5)	90 (40.4)	133 (59.6)	
	Male w/ Minor (<18)				0.15
	Yes	116 (8.1)	40 (34.5)	76 (65.5)	
	No	914 (63.5)	257 (28.1)	657 (71.9)	
Health Status	Self-Reported Health				0.01^†^
	Good or Excellent	1,148 (79.8)	366 (31.9)	782 (68.1)	
	Average	264 (18.3)	84 (31.8)	180 (68.2)	
	Bad or Very Bad	27 (1.9)	16 (59.3)	11 (40.7)	
	Pregnancy Status (among females at birth: n = 668)				0.51
	Yes	23 (5.6)	7 (30.4)	16 (69.6)	
	No	375 (91.7)	158 (42.1)	217 (57.9)	
	Unknown	11 (2.7)	4 (36.4)	7 (63.6)	
	Preexisting Chronic Condition				0.51
	Yes	222 (15.4)	76 (34.2)	146 (65.8)	
	No	1,217 (84.6)	392 (32.1)	825 (67.9)	
	History of COVID				0.06+
	Yes	366 (25.4)	102 (27.9)	264 (72.1)	
	No	1,065 (74.0)	360 (33.8)	705 (66.2)	
	No Response	8 (0.6)	4 (50.0)	4 (50.0)	
Risk Perception	Has taken a COVID test				0.01^†^
	Yes	1,067 (74.1)	365 (34.2)	702 (65.8)	
	No	372 (25.9)	101 (27.2)	271 (72.9)	
Transit Community	Survey Location				0.00*
	Tenosique, Tabasco^Y^	253 (17.6)	84 (33.2)	169 (66.8)	
	Oluta, Veracruz^X^	330 (22.9)	131 (39.7)	199 (60.3)	
	Mexico City^V^	281 (19.5)	110 (39.2)	171 (60.9)	
	Saltillo, Coahuilat	326 (22.7)	73 (22.4)	253 (77.6)	
	Matamoros, Tamaulipas^∞^	249 (17.3)	68 (27.3)	181 (72.7)	

^1^Includes Venezuela, Cuba, and other countries

Statistical significance: * p = 0.00 [CI: 99%]; ^†^ p ≤ 0.05 [CI: 95%]; + p ≤ 0.10 [CI: 90%]

^Y^Entry point: southern border; ^X^Connection point; ^v^Central hub; ^t^Connection point; ^∞^Exit point: northern border

No statistically significant differences were observed between the two groups in terms of age, education level, marital status, having children, ethnicity, traveling with minors, pregnancy status, or preexisting chronic health conditions.

Interviews with individuals vaccinated outside of Mexico revealed two key motivations for vaccination. First, many migrants saw vaccination as essential for border crossings, with one migrant stating, “in Colombia and Brazil, for example, they don’t let anyone leave unless they are vaccinated, even if they are traveling by irregular routes” (09-M-CMOA-V). Second, both migrants and key informants described vaccination as an important protective measure, helping to prevent COVID-19 complications along their journey. As one migrant explained, “In Brazil […] you have to get the shot. I also have to take care of myself and take care of others in the countries I cross into. Because my faith says that the people must come together, they have to help others to get out of this pandemic” (12-M-CMT-TA).

Local key informants also supported the view that migrants’ awareness of vaccination requirements along their journey, particularly for entry into the United States, significantly influenced their vaccine acceptance. One key informant noted, migrants’ main reason for getting vaccinated was the ability to continue their journey” (19-TD-SST-TA). Additionally, another key informant explained, “[…] it depends on the person’s ideology, because even now there are still people that say the virus doesn’t exist […] but most of the population accepts the vaccine […] because it’s a requirement for whenever they want to cross into Mexican territory” (01-TD-OCI-TA).

The multivariate analysis (Table 3) reveals several factors significantly associated with the likelihood of COVID-19 vaccination among migrants in transit through Mexico. Education emerged as a strong predictor, with vaccination rates increasing alongside years of schooling. Migrants with 1–6 years of education had 2.32 times higher odds of being vaccinated than those with no schooling, while those with 15 or more years of education were over three times as likely to be vaccinated (OR = 3.31; 95% CI: 1.89–5.79). This positive association was reinforced by insights from key informants, who noted that migrants from less developed countries, where educational opportunities are limited, often had lower vaccine acceptance: “Usually, most of the migrants we have here are from underdeveloped countries in South America. People that probably don’t have the same opportunity. The level of education that they have is also very low, and all that affects your willingness to be vaccinated.” (18-TD-SSM- TAM).

**Table 3:** Logistic Regression of Sociodemographic, Migration, and Health Factors Associated with COVID-19 Vaccination Among Migrants in Transit Through Mexico, November 2021–May 2022*.

Variable	Adjusted Odds Ratio	*p* value		95% CI	
Education (years)					
No School	1				
1–6 Years	2.32	0.00	1.51		3.55
7–14 Years	2.41	0.00	1.56		3.72
15 and Over	3.31	0.00	1.89		5.79
Last Country of Residence					
Honduras	1				
Guatemala	0.67	0.01	0.48		0.92
Nicaragua	2.6	0.00	1.72		3.91
El Salvador	0.81	0.30	0.54		1.21
Chile	1.89	0.02	1.09		3.29
Other	1.43	0.04	1.01		2.02
Has had COVID					
No	1				
Yes	1.37	0.01	1.08		1.74
Has taken a COVID Test					
No	1				
Yes	4.79	0.00	3.82		6
Survey Location					
Mexico City^V^	1				
Tenosique, Tabasco^Y^	0.47	0.00	0.33		0.67
Saltillo, Coahuila^X^	0.89	0.52	0.62		1.27
Matamoros, Tamaulipas^∞^	0.23	0.00	0.16		0.32
Oluta, Veracruz^X^	2.76	0.00	1.81		4.21

^1^Includes Venezuela, Cuba, and other countries

Statistical significance: * p = 0.00 [CI: 99%]; ^†^ p ≤ 0.05 [CI: 95%]; + p ≤ 0.10 [CI: 90%]

^Y^Entry point: southern border; ^X^Connection point; ^v^Central hub; ^t^Connection point; ^∞^Exit point: northern border

In terms of country of residence before arriving in Mexico, the odds of vaccination were highest among residents of Nicaragua (OR = 2.596; 95% CI: 1.723–3.913) and lowest among residents of El Salvador (OR = 0.807; 95% CI: 0.539–1.208) and Guatemala (OR = 0.666; 95% CI: 0.481–0.921). However, the odds of vaccination were higher among residents of Chile (OR = 1.893; 95% CI: 1.090–3.287) and Other Countries (OR = 1.428; 95% CI: 1.011–2.02) when compared to residents of Honduras. Additionally, a history of COVID-19 (OR = 1.37; 95% CI: 1.081–1.737) and having taken a COVID-19 test (OR = 4.79; 95% CI: 3.825–5.999) were positively associated with vaccination. In terms of survey location (using Mexico City as a reference), the odds of vaccination were highest among respondents from Oluta, Veracruz (OR = 2.761; 95% CI: 1.811–4.209) and lowest among those from Tenosique (OR = 0.470; 95% CI: 0.327–0.674) and Matamoros (OR = 0.225; 95% CI: 0.158–0.321).

Among unvaccinated migrants (n = 916; 38.9% of the total sample), the majority (81.4%) expressed willingness to receive a COVID-19 vaccine (Table 4). Statistically significant differences in acceptance were observed across various sociodemographic and health characteristics. Acceptance was highest among LGBTQIA+ individuals (88.2%), identifying with an ethnic group (82.7%), refugees (88.9%), people reporting good or very good health (83.3%), those who had previously had COVID-19 (86.4%), and those who had taken a COVID-19 test (86.2%). Vaccine acceptance was also higher among participants surveyed in Matamoros, Tamaulipas (88.1%) and among non-pregnant women (86.9%).

**Table 4:** Sociodemographic, Migration, and Health Characteristics of Unvaccinated Migrants in Transit Through Mexico by Willingness to Accept COVID-19 Vaccination, November 2021–May 2022.

Category	Variable	Total (n = 916)	Willing to accept (n = 746; 81.4%)	Unwilling to accept (n = 170; 18.6%)	*p* value
Sociodemographic Characteristics	Age (years): Mean (SD)	26.8 (9.7)	26.9 (9.5)	26.4 (10.6)	0.1
	Education (years): Mean (SD)	7.7 (3.9)	7.7 (3.9)	7.6 (4.1)	0.49
		**n (% of Total)**	**n (% of willing to accept)**	**n (% of unwilling to accept)**	
	Education Level				0.49
	No school	65 (7.1)	52 (80.0)	13 (20.0)	
	1–6 years	362 (39.5)	292 (80.7)	70 (19.3)	
	7–14 years	437 (47.7)	363 (83.1)	74 (16.9)	
	15 and over	52 (5.7)	39 (75.0)	13 (25.0)	
	Sex at Birth				0.13
	Male	657 (71.7)	527 (80.2)	130 (19.8)	
	Female	259 (28.3)	219 (84.6)	40 (15.4)	
	Self-Reported Gender				0.09+
	Male	595 (65.0)	479 (80.5)	116 (19.5)	
	Female	284 (31.0)	236 (83.1)	48 (16.9)	
	LGBTQIA+	34 (3.7)	30 (88.2)	4 (11.8)	
		3 (0.3)	1 (33.3)	2 (66.7)	
	Marital Status				0.31
	Has Partner	355 (38.8)	295 (83.1)	60 (16.9)	
	No Partner	561 (61.2)	451 (80.3)	110 (19.6)	
	Has Children				0.78
	Yes	375 (40.9)	307 (81.9)	68 (18.1)	
	No	541 (59.1)	439 (81.2)	102 (18.9)	
	Ethnic Group				0.03†
	Indigenous	84 (9.2)	66 (78.6)	18 (21.4)	
	Afro-descendent	59 (6.4)	41 (69.5)	18 (30.5)	
	No	773 (84.4)	639 (82.7)	134 (17.3)	
International	Last Country of Residence				0.26
Mobility and Migration	Honduras	609 (66.5)	492 (80.8)	117 (19.2)	
	Guatemala	107 (11.7)	82 (76.6)	25 (23.4)	
	Nicaragua	43 (4.7)	38 (88.4)	5 (11.6)	
	El Salvador	62 (6.8)	56 (90.3)	6 (9.7)	
	Chile	20 (2.2)	16 (80.0)	4 (20.0)	
	Other Countries^1^	75 (8.2)	62 (82.7)	13 (17.3)	
	Migratory Status				0.08+
	Irregular	661 (72.2)	540 (81.7)	121 (18.3)	
	Asylum Seeker	176 (19.2)	135 (76.7)	41 (23.3)	
	Refugee	72 (7.9)	64 (88.9)	8 (11.1)	
	Visa/Permit	7 (0.8)	7 (100.0)	0 (0.0%)	
	Female w/ Minor (<18)				0.3
	Yes	123 (47.5)	107 (87.0)	16 (13.0)	
	No	136 (52.5)	112 (82.4)	24 (17.7)	
	Male w/ Minor (<18)				0.4
	Yes	69 (10.5)	58 (84.1)	11 (15.9)	
	No	588 (89.5)	469 (79.8)	119 (20.2)	
Health Status	Self-Reported Health				0.02^†^
	Good or Excellent	700 (74.4)	583 (83.3)	117 (16.7)	
	Average	10 (1.1)	157 (76.2)	49 (23.8)	
	Bad or Very Bad	206 (22.5)	6 (60.0)	4 (40.0)	
	Pregnancy Status (among females at birth: n = 668)				0.00*
	Yes	18 (7.0)	15 (83.3)	3 (16.7)	
	No	222 (85.7)	193 (86.9)	29 (13.1)	
	Unknown	19 (7.3)	11 (57.9)	8 (42.1)	
	Preexisting Chronic Condition				0.43
	Yes	118 (12.9)	93 (78.8)	25 (21.2)	
	No	798 (87.1)	653 (81.8)	145 (18.2)	
	History of COVID				0.01^†^
	Yes	176 (19.2)	152 (86.4)	24 (13.6)	
	No	727 (79.4)	587 (80.7)	140 (19.3)	
	No Response	13 (1.4)	7 (53.9)	6 (46.2)	
Risk Perception	Has taken a COVID test				0.00*
	Yes	370 (40.4)	319 (86.2)	51 (13.8)	
	No	546 (59.6)	427 (78.2)	119 (21.8)	
Transit Community	Survey Location				0.01^†^
	Tenosique, Tabasco^Y^	344 (37.6)	274 (79.7)	70 (20.4)	
	Oluta, Veracruz^X^	58 (6.3)	40 (69.0)	18 (31.0)	
	Mexico City^V^	80 (8.7)	68 (85.0)	12 (15.0)	
	Saltillo, Coahuilat	216 (23.6)	172 (79.6)	44 (20.4)	
	Matamoros, Tamaulipas∞	218 (23.8)	192 (88.1)	26 (11.9)	

^1^Includes Venezuela, Cuba, and other countries

Statistical significance: * p = 0.00 [CI: 99%]; ^†^ p ≤ 0.05 [CI: 95%]; + p ≤ 0.10 [CI: 90%]

^Y^Entry point: southern border; ^X^Connection point; ^v^Central hub; ^t^Connection point; ^∞^Exit point: northern border

**Fig 2:**
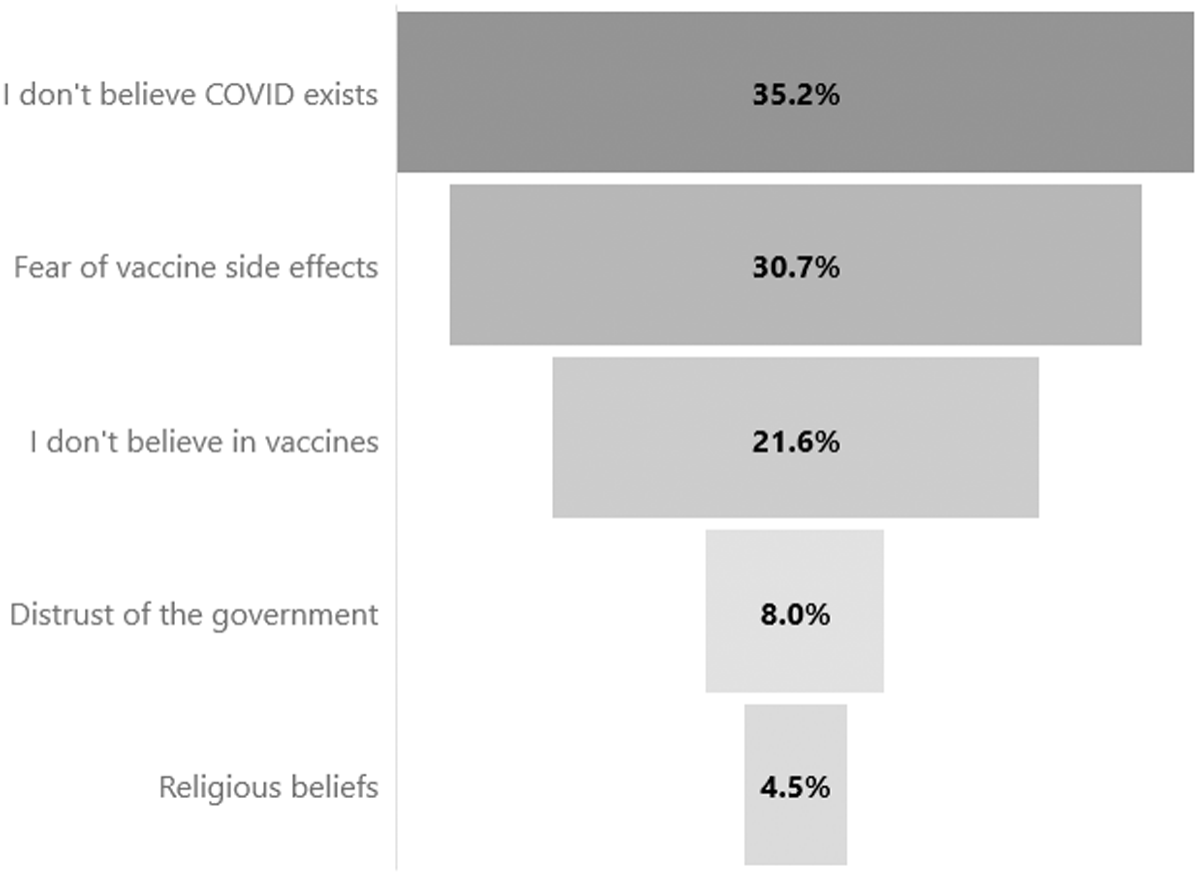
Reasons for COVID-19 Vaccine Refusal Among Unvaccinated Migrants in Transit Through Mexico, November 2021 – May 2022.

The most frequently cited reasons for vaccine rejection in the quantitative survey were disbelief in the existence of COVID-19 (35.2%), fear of vaccine side effects (30.7%), and a general lack of confidence in the vaccine’s safety (21.6%) (Fig 2). Qualitative responses from migrants who refused the vaccine further support these findings. Some migrants expressed a perceived lack of necessity for vaccination, with one respondent asking, “What do I need that [vaccine] for, if I’m not sick? What if that liquid contaminates me or hurts me?” (05-M-CMOA-V). Others shared fears linked to vaccine safety, as one migrant recalled, “I wouldn’t want to get the shot since my cousin died. I’m not sure if he died from it [the vaccine] or if the shot has some kind of chemical…” (03-M-CMOA-V). Key informants reported similar perspectives, observing that some migrants rejected the vaccine due to disbelief in its effectiveness or fear of side effects: “…They say the vaccine doesn’t work, or they’re scared it could cause side effects. They even say COVID itself doesn’t exist…” (04-TD-CMT-TA).

While only a small percentage of survey respondents (4.6%) cited religious beliefs as a reason for vaccine refusal, interviews indicated that religion played a larger role in some communities. Key informants observed that shifting religious practices influenced vaccine hesitancy, particularly among some groups in Guatemala: “… Unfortunately, sometimes Guatemala, has somewhat changed religions; the main religion isn’t Catholicism anymore. . . So, it turns out that, according to them [non-Catholics], God will take care of them and is their main protection . . . And especially in Indigenous communities that are very remote, they won’t get vaccinated” (16-TD-RCG-GUA).

Another factor frequently cited for vaccine hesitancy was the lack of U.S. approval for certain COVID-19 vaccines administered in Mexico, particularly the CanSino vaccine. As one migrant noted, “[…] the population felt uncomfortable with it . . . since it’s not approved by the government [of the United States], not for entering the United States, so migrants saw it as a barrier” (22-TD-OCI). This perspective aligns with a common theme among migrants, who expressed that international recognition—especially from the U.S. and European countries—would influence their acceptance of particular vaccines. One migrant explained, “Actually, down the line, maybe I’ll get one, but I want it to be approved by Europe and the United States governments; because if at some point, I want to go there …” (09-M-CMT-TA). Others expressed a desire to receive specific vaccine doses not currently available, noting, “I haven’t received the second dose because here they [Mexican authorities] don’t have it, and I’m not going to get a different one . . . [vaccination workers] told me to wait, because [the right vaccine] was coming. It was because they had run out, or something.” (09-M- CMOA-V).

## Discussion

This study, conducted in 2021−2022 amid the COVID-19 pandemic, sheds light on the complex factors influencing COVID-19 vaccination rates among migrants in a low- and middle-income country. Mexico’s vaccination coverage for migrants was shaped by both local policy constraints and global vaccine supply factors. Our findings reveal that while local factors directly impacted vaccine accessibility for migrants, global conditions also influenced vaccine acceptability [19] and availability [8] for this population. Though international organizations aimed to protect the most vulnerable through prioritization guidelines, especially age-based criteria [46], these measures created practical barriers for transient migrant populations in Mexico. Migrants often fell outside priority age groups or lacked the documentation required to access vaccination [33,47], creating a gap in vaccination coverage. This gap impacted both Mexico’s national population, with coverage at 66% [47], and, according to our findings, migrants in transit, who had an estimated vaccination rate of 61.1%. This falls below the 66% found by Padhani et al. among migrants in Pakistan [16] but remains above the 45.8% reported by Gubari et al. for migrants in Iraq [15]. Although prioritization strategies based on age and risk factors were essential for protecting high-risk groups, they may have unintentionally limited timely access for many migrant populations.

Among surveyed participants in the vaccinated group, two-thirds reported having received at least one dose of a COVID-19 vaccine before arriving in Mexico, likely reflecting vaccination requirements encountered along their migration route. For instance, interviewed migrants highlighted that in Brazil, vaccination was mandatory for both internal mobility and for leaving the country [48,49]. Additionally, the United States—the primary intended destination for the surveyed migrants—required proof of COVID-19 vaccination for entry [50].

In the vaccinated group, only one-third had been vaccinated within Mexico, a rate that may be tied to national policies. Mexico’s COVID-19 vaccination rollout prioritized older adults and individuals with preexisting health conditions [30], likely limiting vaccine access for in-transit migrants, who tend to be young and without chronic conditions. Although federal guidelines specified that vaccination should be accessible without requiring identification, this policy was not consistently applied; instead, local providers often requested official ID and proof of address, creating significant administrative barriers. Many unvaccinated individuals were irregular migrants who, due to their status, generally lack official documentation or a permanent address. While qualitative data did not reveal one overarching theme, some key informants suggested that migrants without identification faced additional access barriers. Government vaccination websites in Mexico frequently list ID and/or proof of residence as requirements [29,51], possibly disadvantaging irregular migrants and explaining the threefold higher vaccination rate among refugees, who generally possess official documents, compared to irregular migrants in transit.

With respect to gender, our results showed no statistically significant relationship with vaccination, which differs from findings in other studies [52]. However, when analyzing vaccination rates by country of administration, we found that in Mexico, women had a higher vaccination rate than men and LGBTQIA+ individuals. In addition, factors typically associated with healthcare-seeking behaviors, such as caregiving responsibilities—especially relevant for migrant women traveling with children—did not significantly impact vaccination rates and were thus excluded from the adjusted model [53]. Further research is necessary to understand the factors contributing to this variation.

The results of this study also suggest that another factor contributing to lower vaccination coverage within Mexico was the type of vaccine offered. Some migrants reportedly declined vaccines available in Mexico because they were not approved in the United States. Insights from key informant interviews suggest that migrants planning to enter the United States may have postponed vaccination to ensure they could receive a vaccine that met United States entry requirements. As a result, while migrants recognized the protective benefits of vaccination, certain types offered in Mexico did not meet their specific needs, leading some to forgo available doses.

Overall, the factors associated with vaccination coverage among in-transit migrants identified in this study align with patterns described in existing literature on migrant populations [54–57]. This analysis found a significant association between years of education and self-reported vaccination, consistent with other studies that have shown a positive correlation between education level and health-seeking behaviors, including self-care practices [58–60].

Additionally, other factors positively associated with COVID-19 vaccination included previous COVID-19 infection and having undergone COVID-19 testing [61]. These findings may reflect that individuals with a history of infection or testing were more likely to pursue preventive health measures, including vaccination, possibly due to heightened awareness of COVID-19. The observed associations between place of residence, survey location, and differential vaccination coverage could also be linked to vaccine availability and localized management efforts.

The differences in vaccination coverage observed among migrants based on their last country of residence and survey location within Mexico could likely be explained by vaccine availability and local management practices. For example, migrants from Chile, a country that reached its vaccination targets during the study period [62], likely benefitted from broad vaccine access, which may have contributed to the high vaccination rates observed among those arriving from Chile. Within Mexico, local strategies also appeared to play a significant role in influencing vaccination rates. For instance, in Oluta, Veracruz, there were local efforts at migrant shelters, facilitating greater vaccine uptake than in other survey locations [63].

Regarding COVID-19 vaccine acceptability, this study observed a high level of overall acceptability (above 70%). Globally, however, vaccine acceptability varies widely [64], with rates reported as low as 23.6% in Kuwait and as high as 97% in Ecuador [65]. The behavior patterns observed in Ecuador align with the findings of the present study, in which a substantial proportion (81.4%) of unvaccinated migrants, primarily from Latin America and the Caribbean, indicated willingness to receive the vaccine. The key informant interviews further supported this high acceptability rate among migrants, with many seeing the vaccine as a protective measure against the risk of severe complications or death from COVID-19.

When examining COVID-19 vaccine rejection, it’s evident that global misinformation about vaccine efficacy and potential side effects—amplified through social media—polarized public opinion [66]. Our findings suggest that this trend may also have influenced vaccine rejection among migrants in transit through Mexico. Although only a small segment (7.2%) of the total sample (n = 2,355) reported unwillingness to be vaccinated even if it were accessible, this figure is consistent with similar studies [64,67]. The most common reasons cited for vaccine rejection included disbelief in COVID-19 and fears of side effects—findings also noted in studies on Latin American populations [68] and other groups [64]. These factors appear to be influenced by the broader infodemic and misinformation, as reflected in the qualitative insights gathered in this study.

Another reason for vaccine rejection, as reported in the testimonials, was the desire among some migrants to receive the same vaccine brand as their initial dose. This preference likely stemmed from early pandemic vaccination guidelines, which initially emphasized consistency in vaccine type for dosing [69], and although mixed-brand (or hybrid) schedules were later recognized as effective [70], information on these updates may not have reached all migrants in transit. This preference for consistent dosing, therefore, may have contributed to vaccine hesitancy among those who were concerned about mixing brands.

Several limitations should be acknowledged, though they do not detract from the key findings of this study. First, while qualitative insights revealed regulatory and administrative barriers influencing vaccination among in-transit migrants, the quantitative magnitude of these effects could not be determined. One additional factor influencing vaccination rates in Mexico may be the varying duration of stay for migrants in transit; those with shorter stays may have had fewer opportunities to access vaccination, alongside the policy barriers observed. Further areas for exploration include vaccination conditions in other countries, a more detailed breakdown of vaccine coverage (including number of doses and vaccine types), and vaccination status of travel partners.

Finally, because this study was conducted in Spanish, non-Spanish-speaking migrants in transit through Mexico were excluded, which may include some Indigenous groups, individuals from non-Spanish-speaking Latin American countries, and migrants from Africa and Asia. Future studies should facilitate the inclusion of non-Spanish-speaking individuals.

When evaluating the relevance of these findings, it is important to consider both the similarities and unique characteristics of this study population relative to other migrant groups. Additionally, the limitations of using a convenience sample should be acknowledged. While this approach allowed the study to gather a sample that reflects the characteristics of the migrant groups in the surveyed areas, caution is needed when applying these findings to other migrant populations. Shared factors such as mobility, barriers to healthcare access, and communication challenges are common across different migratory contexts, which suggests that some findings may have broader applicability. However, varying migration policies, socioeconomic conditions, and local attitudes toward migration mean that tailored vaccination strategies are needed to meet the unique needs of each migrant group. Therefore, while these findings lay a crucial foundation, they must be carefully contextualized and adapted to ensure their relevance and effectiveness across different migratory settings worldwide.

Furthermore, it is important to note that this study did not apply gender quotas, as its primary focus was on characterizing migratory flows. This may limit our understanding of the full impact of gender on vaccination behaviors. However, even with a low representation of minority ethnic groups, significant differences were observed in vaccination behaviors related to ethnic identity.

At the time this study was conducted, previous research in this area primarily relied on quantitative data from administrative and epidemiological surveillance records collected by health service providers. Qualitative studies, by contrast, often gathered data remotely through telephone calls or videoconferencing platforms such as Zoom [71–73]. This study, however, collected face-to-face primary data directly from migrants, decision-makers, and health service providers. While evidence is still inconclusive regarding the superiority of one data collection method over another [74], the in-person approach of this study may provide additional depth to understanding these populations.

This research was conducted in collaboration with *Casas de Migrantes*, social organizations that, in Latin America and other regions globally, have historically played a crucial role in responding to the diverse needs of migrant populations. The findings generated through this collaboration supported the Casas’ local management efforts and allowed them to identify options for migrant vaccination in alignment with national guidelines.

## Conclusions

Our integrative mixed-methods approach facilitated a deeper understanding of public health phenomena such as vaccination outcomes and associated individual and social determinants. This approach highlights the value of analytical complementarity, where quantitative survey data is combined with qualitative insights from target populations and key informants. Future public health studies should consider this model, leveraging multidisciplinary and transdisciplinary teams to generate, integrate, and analyze comprehensive data to address migrant health needs.

The results of this study  reveal significant disparities in COVID-19 vaccine coverage among migrants in transit through Mexico, often perceived as a uniformly vulnerable group. Differences in vaccination rates were associated with factors such as education level, possession of official documents, history of COVID-19, and history of COVID-19 testing. These findings suggest that future vaccination strategies should consider the distinct needs within the migrant population to target support toward the most vulnerable members of these groups.

Despite the challenges posed by the infodemic, our findings indicate a high acceptability of the COVID-19 vaccine among migrants in transit through Mexico. Beyond the sociodemographic factors mentioned, this acceptability may also be linked to the social value attributed to the vaccine as a means of facilitating international mobility. In this context, it is essential to implement communication strategies that underscore the health benefits of vaccination, emphasizing protections against COVID-19 complications and specific health risks. These strategies could also be valuable in addressing other public health issues sensitive to misinformation, such as the consumption of junk food, excessive alcohol, tobacco, and substances like vapors and hookah, all linked to chronic non-communicable diseases (e.g., hypertension, overweight and obesity, diabetes, and addictions).

Based on these findings, this study provides actionable insights for policymakers and public health authorities. The COVID-19 pandemic revealed the population’s vulnerability to widespread health risks that, in many cases, are preventable through vaccination. Therefore, ensuring a reliable supply of vaccines and the resources for distribution through public health services remains essential. Future mass media campaigns targeting migrant populations should also focus on providing clear, accessible information regarding vaccine safety, efficacy in reducing severe outcomes.

Lastly, the results underscore the need for awareness-raising initiatives and training programs for local decision-makers and healthcare providers to strengthen adherence to established regulatory frameworks. Such measures are necessary to facilitate healthcare access for migrants, including COVID-19 vaccination, ensuring that services are responsive to the unique barriers faced by migrant populations in transit.
